# Topographic Habitat Drive the Change of Soil Fungal Community and Vegetation Soil Characteristics in the Rhizosphere of *Kengyilia thoroldiana* in the Sanjiangyuan Region

**DOI:** 10.3390/jof11070531

**Published:** 2025-07-17

**Authors:** Liangyu Lyu, Pei Gao, Zongcheng Cai, Fayi Li, Jianjun Shi

**Affiliations:** 1 Academy of Animal Husbandry and Veterinary Sciences, Qinghai University, Xining 810016, China; yb230909000074@qhu.edu.cn (L.L.); y200954000466@qhu.edu.cn (P.G.); ys230951310630@qhu.edu.cn (Z.C.); lfy99218@qhu.edu.com (F.L.); 2 Qinghai Province Key Laboratory of Adaptive Management of Alpine Grassland, Xining 810016, China; 3 State Key Laboratory of Ecology and Plateau Agriculture and Animal Husbandry in Sanjiangyuan Jointly Established by the Ministry of Provincial Affairs, Qinghai University, Xining 810016, China

**Keywords:** *Kengyilia thoroldiana*, topographic habitat, fungal community, function prediction, rhizosphere soil, sanjiangyuan region

## Abstract

This study aims to reveal the impact mechanisms of five typical topographic habitats in the Sanjiangyuan region (sunny slope, depression, shady slope, mountain pass, and transitional zone) on the characteristics and functions of rhizosphere soil fungal communities of *Kengyilia thoroldiana*, and to elucidate the association patterns between these communities and soil physicochemical factors. The species composition, diversity, molecular co-occurrence network, and FUNGuild function of microbial communities were investigated based on high-throughput sequencing technology. By combining the Mantel test and RDA analysis, the key habitat factors affecting the structure of the soil fungal community in the rhizosphere zone of *Kengyilia thoroldiana* were explored. The results showed that: ① The composition of the soil fungal community in the rhizosphere of *Kengyilia thoroldiana* in five topographical habitats showed significant differentiation characteristics: the number of OTUs in H2 (depression) and H5 (transitional zone) habitats was the highest (336 and 326, respectively). Habitats H2 showed a significant increase in the abundance of Ascomycota and Mortierellomycota and a significant decrease in the abundance of Basidiomycota compared to the other topographical habitats. ② The diversity and aggregation degree of the soil fungal community in the rhizosphere of *Kengyilia thoroldiana* in five topographical habitats showed differences. ③ Cluster analysis showed that the rhizosphere soil fungi in five topographical habitats of *Kengyilia thoroldiana* could be divided into two groups, with H2, H4 (mountain pass), and H5 habitats as one group (group 1) and H1 and H3 (shady slope) as one group (group 2). ④ The characteristics of the *Kengyilia thoroldiana* community and the physical and chemical properties of rhizosphere soil in five topographical habitats were significantly different, and the height, coverage, biomass, and soil nutrient content were the highest in H2 and H5 habitats, while lower in H1 and H3 habitats, with significant differences (*p* < 0.05). ⑤ Redundancy analysis showed that soil water content was the main driving factor to change the structure and function of the soil fungal community in the rhizosphere of *Kengyilia thoroldiana* in five topographic habitats in the Sanjiangyuan region. This study demonstrated that topographic habitats affected the species composition, functional pattern, and ecosystem service efficiency of the *Kengyilia thoroldiana* rhizosphere fungal community by mediating soil environmental heterogeneity, which provides microbial mechanistic insights for alpine meadow ecosystem protection.

## 1. Introduction

*Kengyilia thoroldiana* is a perennial herb of the genus *Kengyilia* in Poaceae, which is the dominant species in the alpine desert grassland ecosystem [1]. The geographical distribution of *Kengyilia thoroldiana* has obvious high-altitude characteristics, and it is concentrated in the area of 3700–5200 m above sea level in central Asia, showing typical endemic distribution characteristics in the Sanjiangyuan region in China, mainly distributed in Qinghai, Tibet, southeastern Xinjiang, and northern Sichuan provinces [2]. The typical distribution areas in Qinghai Province include the core areas of the Sanjiangyuan region (Tanggula, Qumalai, Maduo, Zhiduo, Zaduo, Nangqian, Yushu, and other counties) and are also distributed around Qinghai Lake, where they are mostly found in hillside fields, valley terraces, and sandy habitats [3]. This species forms a lateral expansion network through the developed drooping rhizome system, and with the deep and wide root structure, it shows outstanding ecological functions in fixing flowing sand and stabilizing surface soils and has become a native grass resource with significant ecological restoration value in the ecological restoration project of the alpine desert grassland in the Sanjiangyuan region [4].

Soil fungi are the core microbial groups that regulate the material cycle and energy flow in soil ecosystems, and their community composition has significant functional diversity, playing crucial roles in key ecological processes such as organic matter mineralization, element cycling and transformation, and humus metabolism [5,6]. As sensitive bioindicators of environmental change, soil fungi not only dynamically reflect soil ecological stability but also reveal the functional differentiation characteristics of microbial communities. The rhizosphere microenvironment is the interface environment of interaction among plant roots, soil, and microorganisms, and its fungal community directly mediates nutrient transformation and substance transport in the rhizosphere through metabolic activities, providing active nutrients for host plants [7]. An in-depth analysis of the species composition and functional characteristics of rhizosphere fungal communities helps clarify the interaction mechanism among plants, microorganisms, and the environment. In recent years, the rapid development of high-throughput sequencing technology has provided technical support for accurately analyzing the structure and functional potential of soil fungal communities. This technology provides deep insights into the species diversity, functional redundancy, and ecological adaptability of fungal communities under complex environmental gradients and has been widely used in environmental microbiome research [8,9,10].

Currently, research on *Kengyilia thoroldiana* mainly focuses on the basic aspects such as morphological descriptions, stress resistance mechanisms (especially cold and drought resistance), and adaptability evaluation of artificial cultivation [1,2,3,4]. However, systematic studies on its resource spatial distribution and rhizosphere fungal community characteristics remain insufficient. If rhizosphere soil fungal community is still insufficient. Although previous work has preliminarily addressed soil fungal diversity in the Sanjiangyuan region [11], investigations into the characteristics of *Kengyilia thoroldiana* rhizosphere fungi—including the coupling relationships between edaphic properties and fungal communities across different topographical habitats—remain unexplored. This study aims to fill the knowledge gap in this field by focusing on the rhizosphere microenvironment of *Kengyilia thoroldiana* across five typical topographical habitats in the Sanjiangyuan region. Employing the Illumina MiSeq high-throughput sequencing platform, we systematically characterize the composition, functional structure, and diversity characteristics of the rhizosphere fungi community, simultaneously measuring soil physical and chemical parameters. Concurrently, revealing the interaction mechanism between fungal communities and the rhizosphere microenvironment. By clarifying the community structure of *Kengyilia thoroldiana* rhizosphere fungi in different topographical habitats and its relationship with soil properties, this study will provide a theoretical basis for the research and development of vegetation restoration technology and the construction of ecological security barriers in the alpine desert grassland ecosystem in the Sanjiangyuan region and expand the research on *Kengyilia thoroldiana* rhizosphere fungi. The hypothesis of this study is that in the Sanjiangyuan region, different topographic habitats (e.g., sunny slope, depression, shady slope, mountain pass, and transition zone) create differentiated rhizosphere soil physicochemical environments (e.g., conductivity, pH, organic carbon content, water content, etc.), which will lead to significant differentiation in the composition, functional structure, and diversity of rhizosphere fungal communities of Kengyilia thoroldiana. Furthermore, there is a significant coupling relationship between fungal community characteristics and soil physicochemical properties.

## 2. Materials and Methods

### 2.1. Study Area Description

**Figure 1:**
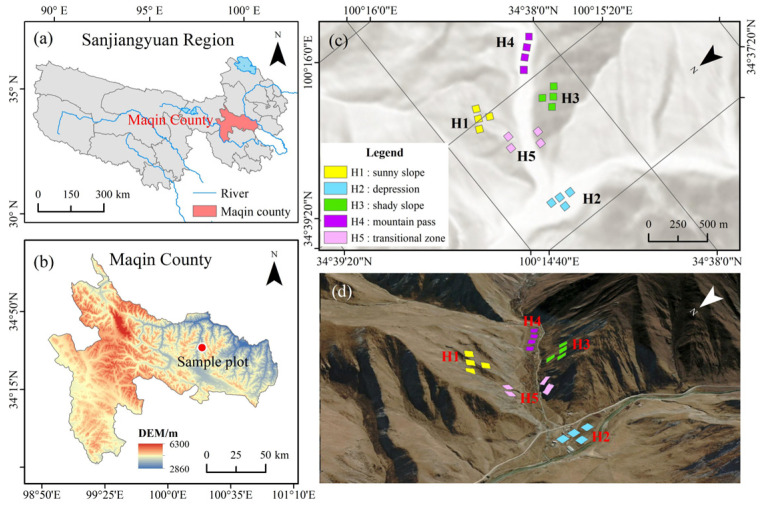
Geographical location of test plot and sampling site. Note: (**a**) Sanjiangyuan region, (**b**) Maqin county, (**c**) geographical location of the sampling point, (**d**) three-dimensional map of the sampling site. The colored squares in (**c**,**d**) represent the transects established within the sample plots.

The experimental area is located in the northeast of Golog Tibetan Autonomous Prefecture in Qinghai Province (33°43′ N~35°16′ N, 98°24′ E~100°56′ E). This region is located within a continental semi-humid plateau climate zone, with an average annual temperature of −3.5 °C and approximately 500 mm of annual precipitation (Figure 1a,b). The ecosystem comprises alpine desert grassland and alpine meadow, with sandy soil, sandy loam, and meadow soil as the primary soil types. The vegetation exhibits distinct alpine characteristics, dominated by Cyperaceae and Gramineae families, with *Carex alatauensis*, *Kengyilia thoroldiana*, and *Poa pratensis* as dominant species [12].

### 2.2. Experimental Design

In July 2023, five types of test plots were established based on topographic features: sunny slope (H1), depression (H2), shady slope (H3), mountain pass (H4), and transition zone (H5) [13]. Four 10 m × 40 m transect were set in each type of plot, and fifteen 0.5 m × 0.5 m measuring quadrats were randomly arranged in each transect (Figure 1c,d). During the peak vegetation growth season, plant community coverage, aboveground biomass, and plant height of *Kengyilia thoroldiana* within each quadrat were systematically measured [14].

### 2.3. Plant Community Investigation and Soil Sample Collection

For two consecutive years (2023, 2024), plant community characterization and rhizosphere soil sampling were conducted concurrently in the peak growth period of pasture grasses in August-September [14]. The sampling contents of the plant investigation include community coverage, aboveground biomass, and plant height determination of *Kengyilia thoroldiana*. Soil sampling adopted a five-point sampling method to obtain 0–2 cm soil from the *Kengyilia thoroldiana* rhizosphere. After thorough mixing and sieving, samples were divided into two portions: one stored at −80 °C for subsequent analysis of soil fungal community diversity. The other is stored at −20 °C for specialized testing of basic soil physicochemical properties, including the contents of organic carbon (SOC), total nitrogen (TN), total phosphorus (TP), and total potassium (TK), soil water content (SWC), electrical conductivity (SEC), pH, and other parameters [15].

### 2.4. Indicator Measurement and Methodology

#### 2.4.1. Vegetation Characterization

The plant height of *Kengyilia thoroldiana* is characterized by the average individual plant height in the quadrat, and the community coverage is estimated by visual inspection. The aboveground biomass was measured by the mowing method: the plants in the quadrat were mowed flush with the ground, oven-dried at 105 °C for 30 min, and then dried at 65 °C for 48 h to constant weight. The aboveground biomass data were obtained by weighing. Each index is expressed by the mean of 15 quadrats [16].

#### 2.4.2. Determination of Physical and Chemical Properties of Soil

Soil electrical conductivity and water content were measured in August 2023 and the same period in 2024 using a TDR350 time domain reflectometer (Spectrum Technologies, El Paso, TX, USA) during morning (8:00–10:00), midday (12:00–14:00), and evening (16:00–18:00) hours under clear weather conditions. Each measurement was repeated three times and monitored continuously for three days [17]. Soil pH was determined by the potentiometric method, organic carbon content via the potassium dichromate oxidation-spectrophotometric method, total nitrogen content using the Kjeldahl method, total phosphorus content through the alkali fusion-molybdenum antimony anti-spectrophotometric method, and total potassium content via the alkali fusion-flame photometer method. Detailed measurement procedures followed Soil Agrochemical Analysis (3rd Edition) [18].

#### 2.4.3. Soil Fungal DNA Extraction, PCR Amplification

Genomic DNA purification was completed by DNA extraction kit, DNA integrity was verified by 1% agarose gel electrophoresis, and DNA samples meeting the quality control standards were sent to a professional sequencing organization for sequencing (Shanghai Majorbio Biotechnology Co., Ltd., Shanghai, China). Amplification sequencing was carried out for the fungal ITS1 variable region, and the PCR amplification system used the classical primer combinations 1737F (5′-GGAAGTAAAAGTCGTAACAAGG-3′) and 2043R (5′-GCTGCGTTCTTCATCGATGC-3′). The amplification products were tested by agarose gel electrophoresis and then mixed and purified in equimolar concentration. The library was constructed according to the standard procedure: flattening the ends of DNA fragments by end-repair enzyme, adding adenylate at the 3′ end, ligating the sequencing junction, and purifying and recovering the target fragments again.

### 2.5. Data Processing

Paired-end sequencing of the MiSeq library was performed on the Illumina MiSeq platform. Following read merging and filtering, denoising of Amplicon Sequence Variants (ASVs) was conducted. The resulting valid data underwent species annotation and abundance analysis to reveal the species composition in the samples. Further soil fungi visualization analyses, including LEfSe (Linear discriminant analysis Effect Size), alpha diversity, beta diversity, FUNGuild functional prediction, and fungal community clustering analysis, were all carried out on the Majorbio Bioinformatics Cloud Platform (https://www.majorbio.com). Raw data organization was performed using Microsoft Excel 2019, and statistical analyses were conducted with SPSS 25.0. Species correlation networks were constructed using Networkx (version 1.11) to calculate interspecies associations. Correlation heatmaps among environmental factors, soil physicochemical properties, and microbial diversity were generated using R 3.5.2. Redundancy analysis (RDA) of environmental factors, soil physicochemical properties, and soil microbial diversity was performed using Canoco 5.0.

## 3. Results

### 3.1. Composition of Soil Fungi Community in Rhizosphere of Kengyilia thoroldiana in Five Topographic Habitats

#### 3.1.1. Quality Analysis of ITS Sequencing Results of Soil Fungi and Changes of OUT Quantity

**Figure 2:**
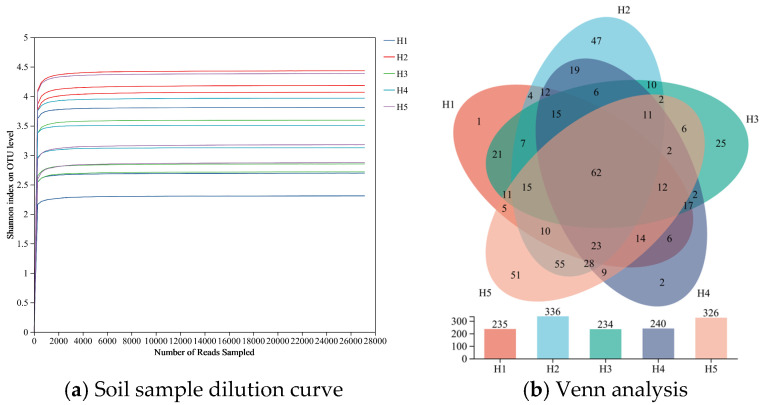
Dilution curve and Venn analysis of soil samples. Note: H1 is a sunny slope, H2 is a depression, H3 is a shady slope, H4 is a mountain pass, and H5 is a transitional zone, the same below. The abscissa represents the amount of sequencing data randomly selected. The ordinate represents the observed Shannon index.

The original double-ended sequences (raw PE reads) were obtained by the Illumina MiSeq sequencing platform, the original tag sequences (raw_tags) were obtained by double-ended sequence splicing and quality filtering, and the high-quality sequences (clean_tags) were obtained by chimera removal and length screening. As illustrated in Figure 2a, the dilution curves of soil samples in the rhizosphere of *Kengyilia thoroldiana* in five types of topographic habitats tend to be flat with the increase of sequencing depth, indicating that the current sequencing amount has fully covered the fungal community, and only a few low-abundance species can be detected by continuing to increase the data amount. Therefore, the sequencing scheme of this study can effectively characterize the true composition characteristics of soil fungal communities in five topographic habitats.

Venn diagram analysis (Figure 2b) revealed that 520 fungal OTUs were detected in the rhizosphere soil of *Kengyilia thoroldiana* in five topographical habitats, among which 235, 336, 234, 240, and 326 OTUs were detected in H1-H5 habitats, respectively, with the order H2 > H5 > H4 > H1 > H3, and the number of OTUs in the H2 habitat was the highest. There were 62 OTUs in 5 habitats, accounting for 11.92%. The number and proportion of OTUs endemic to each habitat are 1 in H1 (0.19%), 47 in H2 (9.04%), 25 in H3 (4.81%), 2 in H4 (0.38%), and 51 in H5 (9.81%).

#### 3.1.2. Analysis of Fungal Community Composition and Relative Abundance

**Figure 3:**
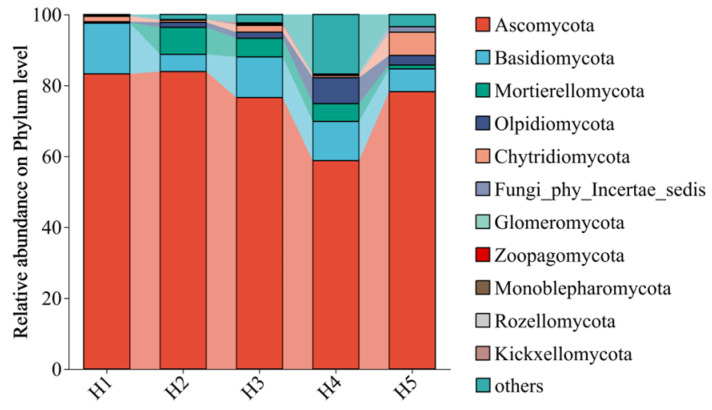
Relative abundance of fungal communities in the rhizosphere soil of *Kengyilia thoroldiana* at the phylum level.

The relative abundance of the top 12 fungi phyla in the rhizosphere soil of *Kengyilia thoroldiana* in five topographical habitats is presented in Figure 3. Among the dominant fungi, Ascomycota (58.77%~83.91%) and Basidiomycota (4.82%~14.29%) occupy an absolutely dominant position, and Mortierellomycota (0.00%~7.65%), Olpidiomycota (0.29%~7.33%), and Chytridiomycota (0.58%~6.53%) are the secondary components. Different topographical habitats have a significant influence on the abundance order of fungi: Compared with H4 habitats, H2 habitats significantly increased the relative abundance of Ascomycota and Mortierellomycota and decreased the abundance of Basidiomycota. Concurrently, the abundance of Olpidiomycota in H2–H5 habitat was higher than in H1.

**Figure 4:**
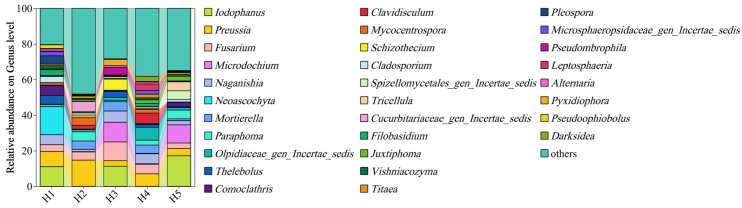
Relative abundance of fungal communities in the rhizosphere soil of *Kengyilia thoroldiana* at the genus level.

The relative abundance of the top 30 fungi genera in the rhizosphere soil of *Kengyilia thoroldiana* in five topographical habitats is shown in Figure 4. The dominant genera include *Iodophanus* (0.00% to 17.18%), *Preussia* (3.11% to 14.73%), *Fusarium* (3.03% to 10.53%), *Microdochium* (0.00% to 11.09%), and *Naganishia* (1.28%~6.31%). The ordering of abundance of genera showed significant differences by habitat, with shaded slopes (H3 habitats) significantly increasing the abundance of *Fusarium*, *Micronodularia*, and *Naganishia* genera compared to sunny slopes (H1 habitats), and a corresponding decrease in the proportion of *Preussia* genera.

#### 3.1.3. LEfSe Analysis of Fungal Community

**Figure 5:**
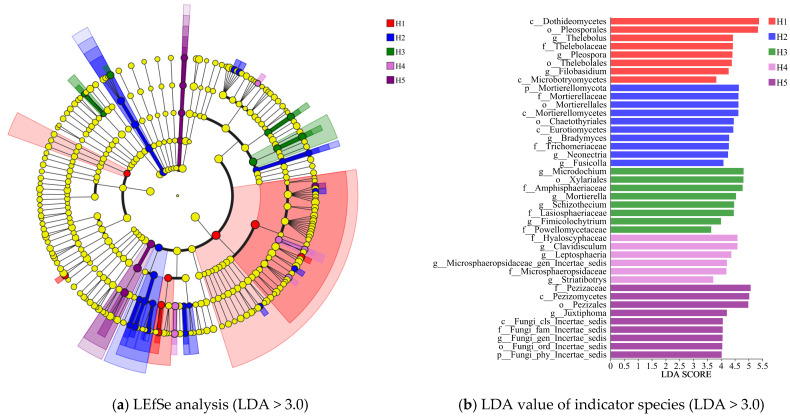
LEfSe analysis of fungal communities in the rhizosphere soil of *Kengyilia thoroldiana*.

LEfSe analysis (Figure 5) screened out the indicator species of the rhizosphere soil fungal community of *Kengyilia thoroldiana* in five topographical habitats through the threshold of LDA >3.0 and detected 41 biomarkers (8 in H1 habitat, 10 in H2 habitat, 8 in H3 habitat, 6 in H4 habitat, and 9 in H5 habitat). The highest scoring markers and their taxonomic levels for each habitat were H1 (c__Dothideomycetes, 5.376), H2 (p__Mortierellomycota, 4.646), H3 (g__Microdochium, 4.816), H4 (f__Hyaloscyphaceae. 4.597), and H5 (f__Pezizaceae, 5.069), and these species significantly influenced the fungal community structure.

### 3.2. Diversity of Soil Fungal Communities in the Rhizosphere of Kengyilia thoroldiana Across Five Topographical Habitats

#### 3.2.1. Analysis of α Diversity of Fungal Communities

**Figure 6:**
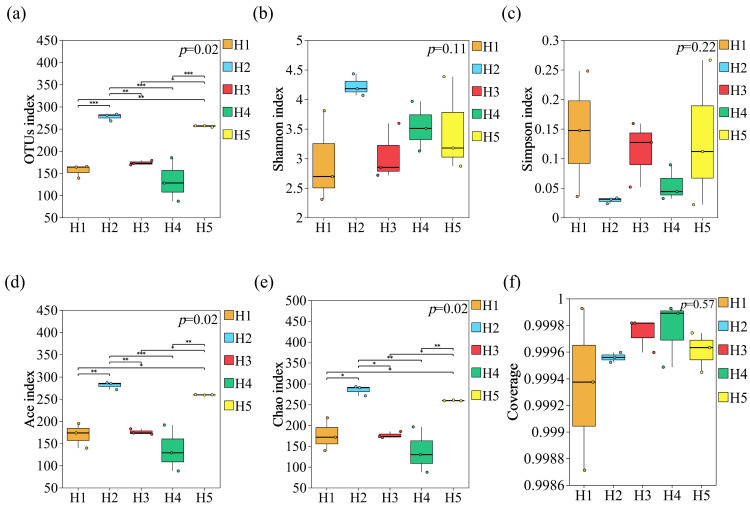
Soil fungal community diversity in the rhizosphere of *Kengyilia thoroldiana*. Note: * represents *p* < 0.05, ** represents *p* < 0.01, and *** represents *p* < 0.001. (**a**) OTUs index; (**b**) Shannon index, (**c**) Simpson index, (**d**) Ace index, (**e**) Chao index, (**f**) Coverage.

As shown in Figure 6a–f, the diversity of soil fungal communities in the rhizosphere of *Kengyilia thoroldiana* in five topographical habitats is significantly different. The number of OTU in H2 habitat was the largest, with 277.33, which was extremely significantly higher than that in H1 habitat, with an increase of 77.78% (*p* < 0.001). The Shannon index reached the maximum value of 4.23 in the H2 habitat, which was 44.48% higher than that in the H1 habitat, but there was no significant difference between them (*p* > 0.05). The Simpson’s index ranked as H1 habitat > H5 habitat > H4 habitat > H3 habitat > H2 habitat, but there was no significant difference among the five habitats (*p* > 0.05). The Ace index (280.93) and Chao1 index (284.74) were the highest in the H2 habitat and the lowest in the H3 habitat (136.03 and 137.74). A highly significant difference in the Ace index (*p* < 0.01) and a significant difference in the Chao1 index (*p* < 0.05) were observed between the H2 and H3 habitats. The Pielou index ranged from 0.58 to 0.75, with the highest in H2 habitat and the lowest in the H1 habitat, with no significant difference between them (*p* > 0.05). These results indicated that both H2 and H5 habitats could increase the number of soil fungal OTUs and elevate the diversity indices of other soil fungal communities, except for the Simpson index.

#### 3.2.2. Analysis of β Diversity (PCoA) of Fungal Communities

**Figure 7:**
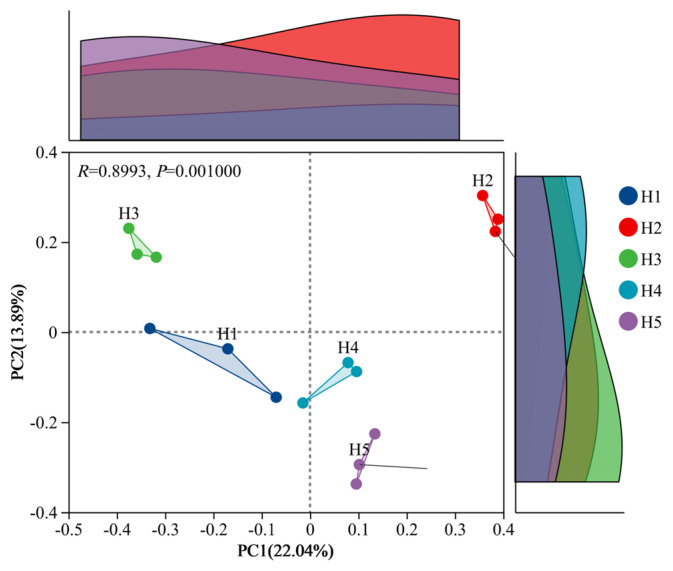
PCoA analysis of rhizosphere soil fungi in *Kengyilia thoroldiana*.

PCoA analysis based on Bray-Curtis distance (Figure 7) showed that there were significant differences in rhizosphere soil fungal communities of *Kengyilia thoroldiana* in five topographical habitats (*p* = 0.001). Among them, H2 and H5 habitats had the closest aggregation (the smallest intra-group variation), and H1 habitats had the highest dispersion (the largest intra-group variation). The main axes, PC1 and PC2, explained the variation of 22.04% and 13.89%, respectively, and the cumulative explanation rate was 35.93%. Fungal communities in different habitats were significantly distinguished on axis PC1, which indicates that this axis is the core factor driving the differentiation of fungal communities in different topographical habitats.

### 3.3. Changes of Single Factor Correlation Network of Soil Fungi in Rhizosphere of Kengyilia thoroldiana in Five Topographical Habitats

**Figure 8:**
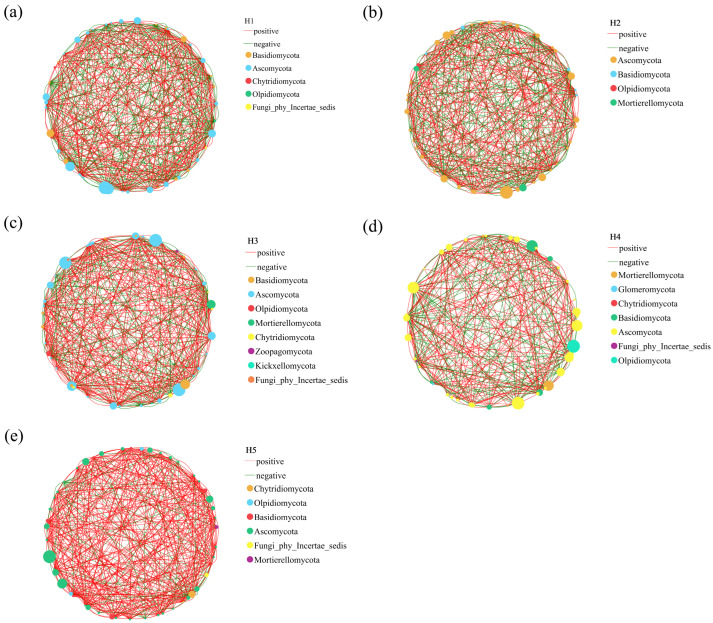
Single-factor correlation network of soil fungal community in *Kengyilia thoroldiana* rhizosphere. Note: Node color and size indicate species type and importance; line color indicates positive or negative correlation, with red positive and green negative; and the number of lines indicates whether the species are closely related. (**a**) sunny slope, (**b**) depression, (**c**) shady slope, (**d**) mountain pass, (**e**) transitional zone.

Based on the horizontal single-factor correlation network (Figure 8), it was found that the network topological characteristics of soil fungal communities in the rhizosphere of *Kengyilia thoroldiana* in five topographical habitats were different; that is, the network structural characteristics of soil fungal communities in the rhizosphere of *Kengyilia thoroldiana* in different topographical habitats showed significant differences. Habitat H4 had the lowest number of network edges (311), which is 28.67 percent less than the number of network edges in habitat H2 (412); there were little differences in the number of edges and nodes of the five terrain and habitat networks; the positive network correlation was highest for Habitat H5 (84.24%), followed by Habitat H3 (66.75%), and the positive network correlation for all five habitats exceeded 50.00%. In the fungal unifactorial correlation network, the key connecting nodes varied by habitat: the important nodes in the H1 habitat were Basidiomycota, Ascomycota, and Chytridiomycota. Habitat H2 important nodes were Ascomycota, Basidiomycota, and Olpidiomycota. Habitat H3 significant nodes were Basidiomycota, Ascomycota, and Olpidiomycota. Habitat H4 significant nodes were Mortierellomycota, Glomeromycota, and Chytridiomycota. The important nodes of the H5 habitat are Chytridiomycota, Olpidiomycota, and Basidiomycota.

### 3.4. Prediction on the Function of Soil Fungal Community in Kengyilia thoroldiana Rhizosphere in Five Topographic Habitats

**Figure 9:**
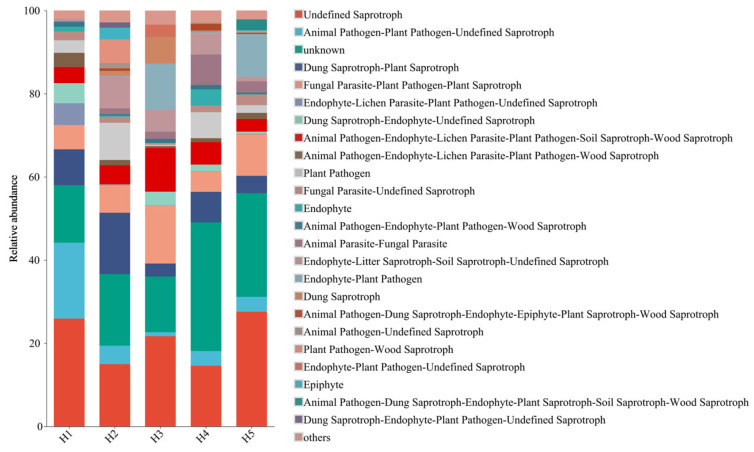
Heatmap of FUNGuild function prediction of *Kengyilia thoroldiana* rhizosphere soil fungal community.

Based on FUNGuild functional classification, the functions of the soil fungal community in the rhizosphere of *Kengyilia thoroldiana* in five topographic habitats were predicted (Figure 9). A total of 24 nutritional types were identified; the top five nutritional types were Undefined Saprotroph, Animal Pathogen-Plant Pathogen-Undefined Saprotroph, Unknown, Dung Saprotroph-Plant Saprotroph, and Fungal Parasite-Plant Pathogen-Plant Saprotroph. Among them, the fungi in habitat H1 are mainly undefined saprotroph, with a relative abundance of 25.91%, followed by Animal Pathogen-Plant Pathogen-Undefined Saprotroph and Unknown, with relative abundances of 18.27% and 13.81%, respectively. The relative abundance of Unknown fungal in habitat H2 is the highest, accounting for 17.12%, followed by Undefined Saprotroph and Dung Saprotroph-Plant Saprotroph, with relative abundances of 14.98% and 14.78%. Undefined Saprotroph is the main fungus in habitat H3, with relative abundance reaching 21.71%, followed by Fungal Parasite-Plant Pathogen-Plant Saprotroph and Unknown, with relative abundances of 13.95% and 13.40%. The relative abundance of unknown fungal taxa in Habitat H4 was the highest, accounting for 30.93%, followed by Undefined Saprotroph and Dung Saprotroph-Plant Saprotroph, with relative abundances of 14.60% and 7.35%. Undefined Saprotroph accounted for the highest proportion of fungi in habitat H5, with a relative abundances of 27.58%, followed by Unknown and Fungal Parasite-Plant Pathogen-Plant Saprotroph, with relative abundances of 24.94% and 9.98%, respectively. Compared with habitat H1, the other four topographical habitats can reduce the abundance of Animal Pathogen-Plant Pathogen-Undefined Saprotroph fungi.

### 3.5. Cluster Analysis of Soil Fungal Community in Kengyilia thoroldiana Rhizosphere in Five Topographic Habitats

**Figure 10:**
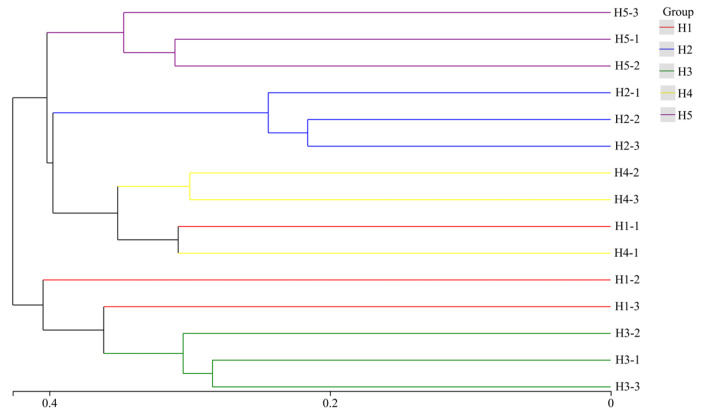
Cluster analysis of soil fungal community in *Kengyilia thoroldiana* rhizosphere.

Based on hierarchical clustering distance, 15 soil samples of *Kengyilia thoroldiana* rhizosphere in five topographic habitats were clustered and analyzed. As shown in Figure 10, rhizosphere soil fungi in five topographic habitats of *Kengyilia thoroldiana* were divided into two groups: H2, H4, and H5 habitats as one group (group 1) and H1 and H3 habitats as another group (group 2). At a distance of 0.41, Group 1 was divided into two subgroups, with Habitat H5 alone as one subgroup and Habitats H2 and H4 as the other subgroup; Group 2 was also divided into two subgroups, with Habitat H1 as one subgroup and Habitat H3 as the other. In addition, three soil samples from habitats H1, H2, H3, and H5 were in the same branch, suggesting that these habitats have a high degree of similarity in the structure of the soil fungal community, while three soil samples from habitat H4 were distributed in different branches, suggesting that the composition of their soil fungal communities showed significant variation.

### 3.6. Changes of Vegetation and Soil Characteristics of Kengyilia thoroldiana Community in Five Topographic Habitats

Table 1 shows significant differences in plant community coverage, biomass, and plant height of *Kengyilia thoroldiana* in five topographical habitats. The community coverage ranged from 35.11% to 70.81%, and the order from highest to lowest was H2 habitat > H5 habitat > H4 habitat > H3 habitat > H1 habitat, among which H2 and H5 habitats had the highest coverage, with 70.81% and 70.25%, respectively. There was no significant difference between them, but they were significantly higher than the H1 habitat (*p* < 0.05). The biomass in the H2 habitat reached the peak of 652.49 g·m^−2^, which was 88.47% higher than that in the H1 habitat (346.21 g·m^−2^), with a significant difference (*p* < 0.05). Plant height exhibited a spatial pattern analogous to biomass distribution. The plant height in the H2 habitat was 34.87 cm, which was 1.87 times that in the H1 habitat (18.63 cm), with a significant difference (*p* < 0.05). In summary, topographic habitats had significant regulatory effects on cover development, material accumulation, and morphogenesis of *Kengyilia thoroldiana* communities, with H2 habitats showing optimal ecological adaptations.

Table 1 also reveals significant differences in the physical and chemical properties of *Kengyilia thoroldiana* rhizosphere soil in five topographical habitats (Table 1). The specific phenotype is that the soil water content is between 23.13% and 40.38%, and the order from highest to lowest is H2 habitat, H5 habitat, H4 habitat, H3 habitat, and H1 habitat, in which the water content of H2 habitat is 74.58% higher than that of H1 habitat. Soil conductivity was greatest the in H1 habitat, up to 1098.36 μs·cm^−1^, and smallest in the H2 habitat, only 599.41 μs·cm^−1^, representing a 45.43% decrease (*p* < 0.05). Soil pH ranged from 7.97 to 8.66 and was weakly alkaline. H2 habitats exhibited significantly lower pH than H1, H3, and H4 habitats. Soil organic carbon content ranged from 5.60 to 15.30 g·kg^−1^ and reached 15.30 g·kg^−1^ in H2 habitat, an increase of 173.21% compared to H1 habitat (*p* < 0.05), which was significant (*p* < 0.05). The soil total nitrogen content was the highest in the H2 habitat, which was 1.55 g·kg^−1^, which was 101.30% higher than that in the H1 habitat, with a significant difference (*p* < 0.05). Soil total phosphorus content reached 0.58 g·kg^−1^ in H2 habitat, which was 81.25% higher than that in H1 habitat, and this difference was significant (*p* < 0.05). Soil total potassium content ranged from 19.08 to 25.28 g·kg^−1^, with H2 habitat containing the highest level at 25.28 g·kg^−1^, followed by H5 habitat with 22.05 g·kg^−1^. In conclusion, H2 habitat has obvious advantages in soil water conservation, salt leaching, and nutrient accumulation, and its physicochemical properties are more conducive to the material cycle and ecological function maintenance of the *Kengyilia thoroldiana* community.

### 3.7. Coupling Relationships Between Soil Environmental Factors, Plant Community Characteristics, and the Diversity of Soil Fungal Community in Kengyilia thoroldiana Rhizosphere

**Figure 11:**
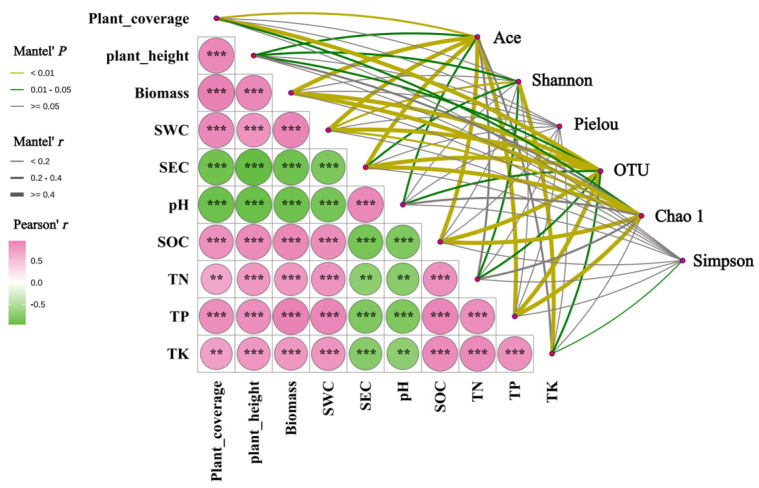
Mantal analysis of soil physical and chemical properties, plant community characteristics, and soil fungal community diversity in *Kengyilia thoroldiana* rhizosphere. Note: ** denotes *p* < 0.01, *** denotes *p* < 0.001; SWC: Soil water content; SEC: Soil electrical conductivity; SOC: Soil organic carbon; TN: Soil total nitrogen; TP: Soil total phosphorus; TK: Soil total potassium.

By using the Mantel test method, the correlation between three plant community indexes, seven soil physicochemical indexes, and the soil fungal community diversity in *Kengyilia thoroldiana* rhizosphere was analyzed, and the results are shown in Figure 11. There was a highly significant positive correlation (*p* < 0.001) between SEC and pH, while both showed highly significant (*p* < 0.01) or highly significant negative correlations (*p* < 0.001) with the other three plant indicators and five soil physicochemical indicators. There were highly significant (*p* < 0.01) or extremely significant positive correlations (*p* < 0.001) between the three plant indicators and the other five soil indicators.

The Ace index of soil fungi in the rhizosphere of *Kengyilia thoroldiana* showed significant (*p* < 0.05) or highly significant correlation (*p* < 0.01) with three plant indexes and five soil indexes except TN and TK. The Ace index of soil fungi in the rhizosphere of *Kengyilia thoroldiana* showed significant (*p* < 0.05) or highly significant correlation (*p* < 0.01) with three plant indexes and five soil indexes except TN and TK. The Shannon index showed highly significant correlations (*p* < 0.01) with SWC and TK and significant correlations (*p* < 0.05) with plant coverage, SEC, and TN. Significant (*p* < 0.05) or highly significant (*p* < 0.01) correlations were found between the number of OTUs and all three plant indicators and seven soil indicators. The Chao1 index was highly significantly correlated (*p* < 0.01) with biomass, SWC, SEC, SOC, and TP and significantly correlated (*p* < 0.05) with plant coverage. There was a significant (*p* < 0.05) correlation between Simpson’s index and TK only.

**Figure 12:**
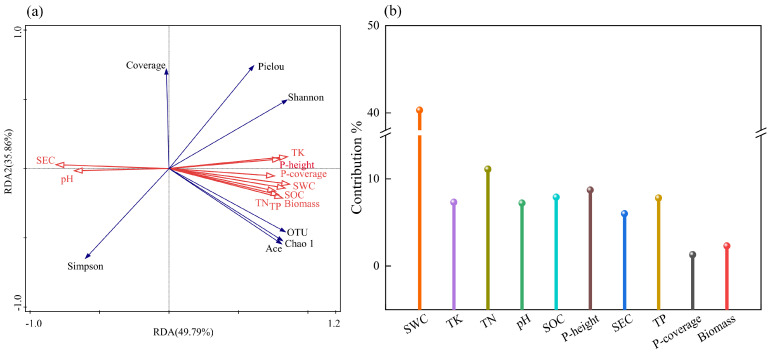
RDA analysis of soil physical and chemical properties, plant community characteristics, and soil fungal community diversity in *Kengyilia thoroldiana* rhizosphere. Note: (**a**) Red arrows indicate plant community characteristics and soil factors, and blue arrows indicate fungal diversity indices. (**b**) SWC: Soil water content; SEC: Soil electrical conductivity; SOC: Soil organic carbon; TN: Soil total nitrogen; TP: Soil total phosphorus; TK: Soil total potassium. P-Coverage: plant coverage; P-height: plant height.

RDA analyses were performed with soil fungal community structure as the response variable and plant characteristics and soil characteristics as explanatory variables, respectively, and the results are shown in Figure 12. Soil fungal community structure with plant characteristics and soil properties explained 49.79% and 35.86% in axes I and II. Soil water content (SWC) had the longest connecting arrow with an explanatory rate of 38.1% and a contribution rate of 40.3% and reached a significant level (*p* = 0.002), indicating that it is the dominant environmental factor influencing the structure of fungal communities. SEC and pH were positively correlated with Simpson’s index and negatively correlated with plant coverage, Pielou’s index, Shannon’s index, OTU number, Chao1 index, and Ace index. The remaining plant community characteristics and soil physicochemical properties were positively correlated with all soil fungal diversity indices except Simpson’s index. These results indicate that soil moisture conditions have a decisive influence on the distribution of fungal communities, while soil conductivity and pH indirectly regulate the diversity pattern of fungal communities by shaping the environmental stress conditions. This synergistic effect of multi-scale environmental factors has jointly driven the succession process of underground fungal communities.

## 4. Discussion

### 4.1. Changes of Physicochemical Properties of Kengyilia thoroldiana Vegetation and Rhizosphere Soil in Five Topographic Habitats

Soil organic carbon, nitrogen, phosphorus, potassium, and other components, together with soil pH, electrical conductivity, and water content, constitute critical components of the soil quality control system [19]. The special pattern of topographic differentiation in the Sanjiangyuan region leads to significant microenvironmental differences in the habitat of *Kengyilia thoroldiana*, and this topography-driven effect is particularly evident in soil-vegetation system interactions [20]. Different topographical habitats show unique soil development patterns: H1 and H3 habitats have strong wind erosion due to surface exposure, and their soil nutrient retention capacity is weaker than that of H2 habitats and H5 habitats; the permeability of H4 habitats decreased due to the development of surface soil crust, which inhibited evaporation to some extent, but the degradation of soil structure limited the nutrient cycle efficiency. Comparatively speaking, H2 and H5 habitats show better characteristics of soil-vegetation co-evolution with higher vegetation coverage (over 65%) and aboveground biomass forming effective ecological barriers, and the continuous input of litter significantly improves the content of organic matter and mineral nutrients in rhizosphere soil. This vegetation-soil positive feedback mechanism is particularly significant in slope gradient. With the steep slope decreasing, soil nutrients show a significant cumulative effect, and plant community productivity is also significantly enhanced [21]. The HunShanDake sparse forest grassland system also showed a soil nutrient distribution pattern of depression > shady slope > sunny slope, with flat habitats having higher nutrient supply capacity and plant productivity compared to slopes [21]. In this study, the depression habitat (H2) is a typical unit with a low slope, and the high vegetation coverage and biomass contribute to the enrichment effect of soil nutrients, which is consistent with the previous theory of terrain-driven soil vegetation development [21]. This phenomenon may be due to the fact that vegetation restoration improves soil structure through canopy interception and root sequestration, creating a mechanism for organic matter stabilization, an ecological service that is particularly prominent in habitats with slower slopes (H2 and H5) [21,22]. Through regulating microclimate, hydrological processes, and surface erosion intensity, topography shapes the spatial differentiation pattern of soil nutrients. Future studies can further quantify the turnover efficiency of soil nutrients in different topographic units and their vegetation response thresholds so as to provide a more accurate theoretical basis for the restoration of degraded grasslands in different topographic habitats.

### 4.2. Changes of Soil Fungal Community Structure and Function in Rhizosphere of Kengyilia thoroldiana in Five Topographical Habitats

There are significant differences in the soil microenvironment of five typical topographical habitats in the Sanjiangyuan region, and this habitat heterogeneity significantly shapes the composition and structure of the soil fungal community in the rhizosphere of *Kengyilia thoroldiana*. In this study, the number of fungal OTUs in the H2 and H5 habitats was 336 and 326, respectively, which was significantly higher than that in the H1 and H3 habitats. It is hypothesized that the H2 and H5 habitats were located between the H1 and H3 habitats and the terrain was gentle, so the winds and runoff brought the litter, soils, and fungi attached to them from the H1 and H3 habitats into the H2 habitats to achieve the secondary colonization, which increased the number of soil fungi in the H2 habitats [23]. Meanwhile, the study found that the number of fungal OTUs in H3 and H4 habitats was slightly higher than that in H1 habitats. This may be attributed to the fact that H3 habitats, characterized by weaker light intensity, higher soil moisture content, and greater accumulation of litter, offer more suitable microhabitats for fungi, resulting in a higher number of fungal OTUs compared to H1 habitats [24]. At the phylum-level taxonomic unit, Ascomycota, Basidiomycota, and Mortierellomycota hold an absolute dominance in the rhizosphere soil of *Kengyilia thoroldiana* with a combined relative abundance exceeding 70%, indicating that these three fungal groups play central roles in soil ecological processes within this region [25]. Notably, the relative abundance of Ascomycota in depression habitats was significantly higher than in mountain pass habitats, which is closely associated with the rich organic matter content in these habitats [26]. Members of the Ascomycota generally possess potent lignocellulose-degrading abilities and play a pivotal role in the decomposition process of the abundant litter input in H2 habitats [26]. In contrast, the Basidiomycota demonstrates higher ecological adaptability in nutrient-poor H1 habitats, with its oligotrophic characteristics providing it with a competitive edge in resource-limited environments [27]. The aforementioned findings corroborate the “terrain-soil-microbe” cascade effect theory, which posits that terrain, by shaping gradients of soil physicochemical properties, drives niche differentiation within fungal communities [23,24,25,26,27].

The research findings indicate that there is a significant differentiation in α diversity of fungal communities in the rhizosphere soil of *Kengyilia thoroldiana* across five types of topographic habitats in the Sanjiangyuan region. The number of fungal OTUs, as well as the Ace and Chao1 indices in H1 habitats, are significantly lower compared to those in H2 habitats (*p* < 0.05). The reason for this phenomenon may be influenced by soil physicochemical properties (water content, pH, conductivity, and soil nutrients) and may also be related to plant community characteristics [28,29]. Based on PCoA analysis, it was found that the beta diversity (between-habitat diversity) of soil fungal communities in different topographic habitats also showed characteristic distribution patterns. Among them, samples from the H2 and H5 habitats exhibited close clustering in the ordination space, whereas samples from the H1 and H3 habitats showed a dispersed distribution. It was inferred that this was also associated with the soil physicochemical properties and plant community characteristics of topographic habitats. The soil pH, water content, nitrogen (N), phosphorus (P), potassium (K) contents, and plant community characteristics varied considerably in the H1 and H3 topographic habitats, compared to the H2 and H5 habitats, which showed a higher degree of homogeneity [28,29]. This finding is highly consistent with theoretical model proposed by Dong et al. [30] on the construction of fungal communities driven by environmental factors, in which a multidimensional combination of environmental variables determines the changes in species diversity of fungal communities through the mechanisms of resource allocation and ecological niche differentiation.

The interaction network among members of soil fungal communities serves as an essential dimension for elucidating their community structure and functions [31]. In this study, network analysis was used to reveal the co-occurrence network patterns of soil fungal communities in the rhizosphere of *Kengyilia thoroldiana* in five topographical habitats. The results showed that the fungal network in the H5 habitat exhibited the strongest positive correlation, indicating that the fungal groups in this habitat tended to form cooperative relationships such as mutualism and commensalism. This positive interaction network enhanced the efficiency of nutrient cycling and promoted the ability of plants to acquire soil resources, which is consistent with the previous research conclusions on the regulation of fungal interaction mode by habitat conditions [32]. According to the network topology analysis, Basidiomycota and Ascomycota were identified as the key groups of the rhizosphere soil fungal community of *Kengyilia thoroldiana* in the Sanjiangyuan region, and their node centrality index is significantly higher than other groups. It indicates that these two types of fungi play a central function in ecological processes such as organic matter decomposition and nutrient transformation, which can directly affect community stability and ecosystem service functions [33]. Based on the cluster analysis of soil fungal community structure in the rhizosphere of *Kengyilia thoroldiana*, the results showed that the habitats H2, H4, and H5 were clustered into one group (group 1), while the habitats H1 and H3 belonged to another group (group 2). This grouping pattern was highly coupled to the soil physicochemical gradient: soil water content was significantly higher in habitats H2 and H5 (40.38% and 35.43%) than in H1 and H3 (28.72% and 30.14%), which also showed a clear advantage in terms of soil nutrient content (carbon, nitrogen, phosphorus, and potassium). Moisture, as a key environmental factor, not only directly affects fungal metabolic activities but also indirectly shapes community composition by regulating litter decomposition rate [34]. This study further corroborates that the spatial heterogeneity of soil moisture and nutrients drives the adaptive differentiation of fungal communities by modulating fungal interaction patterns and the abundance of key taxonomic groups.

Different topographical habitats have driven the functional differentiation of soil fungal communities in *Kengyilia thoroldiana* rhizosphere by shaping unique microenvironments [35]. The function prediction based on FUNGuild shows that the fungal community is mainly composed of three functional groups: saprotrophic, pathogenic, and symbiotic, among which saprophytic is dominant. This kind of fungi decomposes animal and plant residues by secreting extracellular enzymes and accelerates the process of organic matter mineralization. Its dominant position is closely related to the large amount of litter input in the alpine meadow ecosystem in the Sanjiangyuan region rich carbon source substrates provide the energy basis for its metabolic activities and at the same time promote the soil nutrient cycle [36]. It is worth noting that the abundance of pathological nutritional fungi in the H1 habitat is significantly higher than that in other habitats, which is closely related to the low vegetation coverage, many poisonous weeds, and poor soil in this habitat [37]. Poor habitat conditions limit the colonization ability of saprophytic fungi and indirectly create ecological niche space for pathogenic fungi [37]. At the same time, it was found that there were a certain proportion of unidentified/undefined functional groups in all habitats, which reflected the complexity of the function of rhizosphere soil fungi in *Kengyilia thoroldiana* and the lack of research depth. In the future, it will be necessary to further analyze the ecological functions of these “dark matter” fungi, especially their adaptive mechanisms in extreme environments, by combining metagenomics technology.

### 4.3. Correlation Analysis of Soil Fungi Composition in Kengyilia thoroldiana Rhizosphere with Soil Physical and Chemical Properties and Plant Community Characteristics

The Mantel test revealed that there was a significant vegetation-soil-microorganism mutualistic feedback network in the *Kengyilia thoroldiana* ecosystem in the Sanjiangyuan region. Specifically, plant height, community cover, and biomass showed strong positive coupling with fertility indicators such as soil water content, organic carbon, and total nitrogen, and negative correlations with physicochemical parameters such as soil conductivity and pH. This pattern is highly consistent with the findings of Gao Pei et al. [38] on the stability of alpine meadow ecosystems, that is, the enhancement of soil nutrient supply capacity and water effectiveness is the basis for the healthy development of plant communities. The diversity indices (Chao1 index, Ace index, and number of OTUs) of soil fungal communities, as a key link in this mutual feedback network, showed significant positive correlation with vegetation cover and biomass. This suggests that vegetation restoration not only promotes fungal colonization by improving the microenvironment, but also its root secretions are more likely to provide ecological niches for specific fungal taxa [39]. Conversely, the fungal community feeds back to the plant through nutrient mineralization, disease suppression, and other functions, forming a synergistic vegetation-soil-microbe evolutionary cycle [40]. Soil moisture content plays a central regulatory role in this feedback system. Soil hydrological differences induced by topographical gradients significantly shape fungal community structure: in H1 habitats, poor water permeability arises from soil compaction and high bulk density, whereas in H2 habitats, abundant litter input enhances soil water-holding capacity, thereby promoting organic matter accumulation and fungal diversity [41]. This moisture-driven fungal community succession further validates the pivotal role of soil physico-chemical properties in microbially mediated ecosystem functioning [42]. The ecological restoration mechanisms revealed in this study in the Sanjiangyuan region indicate that improving soil hydrological conditions and fertility levels through the reestablishment of *Kengyilia thoroldiana* vegetation can simultaneously achieve microbial community optimization and enhancement of ecosystem multifunctionality, providing a scientific basis for ecological restoration of alpine degraded meadows.

## 5. Conclusions

The response of the rhizosphere fungal community of *Kengyilia thoroldiana* to five topographical habitats showed significant differentiation: the transition zone habitat harbored the highest number of endemic fungi, while the depression habitat enriched the highest number of fungi. α-diversity indices (OTU count, Ace index, and Chao1 index) were significantly higher in the depression and transition zone habitats compared to the sunny and shady slopes (*p* < 0.05). Network topology analysis revealed that fungal community networks in the depression, shady slope, and transition zone habitats exhibited higher complexity and stronger positive correlations. Hierarchical clustering grouped the five habitats into two clusters (Cluster 1: depression, mountain pass, transition zone; Cluster 2: sunny slope, shady slope). Functional prediction indicated that, except for the sunny slope, the other four habitats reduced the abundance of animal pathogen-plant pathogen-undefined saprotroph groups.

There are significant differences in the physical and chemical properties of soil in the rhizosphere of *Kengyilia thoroldiana* in five types of topographic habitats. The soil water content and nutrient indexes (organic carbon, total nitrogen, total phosphorus, and total potassium) in depression and transitional zone habitats are significantly higher than those in sunny and shady slopes, and the coverage, biomass, and plant height of the *Kengyilia thoroldiana* community are also higher. Redundancy Analysis (RDA) revealed that soil water content is the core environmental factor driving rhizosphere soil fungal community succession in five topographical habitats of *Kengyilia thoroldiana* in the Sanjiangyuan region (*p* = 0.002). It not only directly enhances fungal diversity but also indirectly influences fungal metabolic functions by regulating soil nutrient content.

## Figures and Tables

**Table 1:** Characteristics of the *Kengyilia thoroldiana* community and soil environmental factors in five types of topographic habitats.

Treatment	Plant Coverage/(%)	Plant Height/ (cm)	Biomass/(g·m^−2^)	SWC/(%)	SEC/ (μs·cm^−1^)	pH	SOC/ (g·kg^−1^)	TN/ (g·kg^−1^)	TP/ (g·kg^−1^)	TK/ (g·kg^−1^)
H1	35.11± 3.9c	18.63± 2.34d	346.21± 20.97d	23.13± 3.34c	1098.36± 63.67a	8.66± 0.05a	5.60± 0.46d	0.77± 0.16d	0.32± 0.02b	19.08± 2.12c
H2	70.81± 6.82a	34.87± 1.65a	652.49± 20.65a	40.38± 4.16a	599.41± 38.50c	7.97± 0.12c	15.30± 1.53a	1.55± 0.07a	0.58± 0.06a	25.28± 0.82a
H3	44.05± 2.69c	24.18± 1.60c	395.31± 30.94c	27.34± 2.85c	932.54± 120.11b	8.35± 0.02b	5.84± 0.25d	1.06± 0.14b	0.36± 0.05b	20.00± 0.46bc
H4	56.12± 6.14b	28.16± 2.07b	468.17± 37.23b	27.83± 1.27c	832.02± 80.23b	8.23± 0.03b	8.43± 0.37c	0.82± 0.02d	0.39± 0.03b	20.01± 1.21bc
H5	70.25± 4.29a	31.69± 1.36a	623.71± 10.64a	35.43± 3.23b	648.72± 62.92c	8.08± 0.06c	10.75± 0.60b	1.20± 0.08b	0.52± 0.01a	22.05± 1.41b

Note: Data are presented as mean ± standard deviation (SD). Different lowercase letters within the same column indicate significant differences at the 0.05 level (*p* < 0.05). SWC: Soil water content; SEC: Soil electrical conductivity; SOC: Soil organic carbon; TN: Soil total nitrogen; TP: Soil total phosphorus; TK: Soil total potassium.

## Data Availability

The original contributions presented in this study are included in the article; further inquiries can be directed to the corresponding author.
